# Still a hopeless case for personalized oncology? Pancreatic cancer revisited

**DOI:** 10.18632/oncoscience.464

**Published:** 2018-08-22

**Authors:** Wilko Weichert, Martin R. Sprick, Jens T. Siveke

**Affiliations:** Member of the German Cancer Consortium (DKTK), Institute of Pathology, Technical University Munich, 81675 Munich, Germany

**Keywords:** pancreatic cancer, personalized oncology, ge-nomics, RNA subtypes, prognosis

In many cancer entities, morphomolecularly informed, individually tailored, targeted treatment has strongly improved patient outcome. Recent advances in immune oncology in concert with novel targeted molecular agents even brought pharmacological “cure” within reach for a considerable number of patients with metastatic solid tumors. However, as always in the last decades, one of the large tumor entities is left behind: pancreatic cancer.

But is this perception really true? Indeed, a plethora of – mostly unselected - molecularly targeted therapeutic approaches have failed to show clinical activity in PDAC. And, yes, if only mutations are taken into account the genome of PDAC is unnervingly dull and stable [1] and those mutations which are uniformly present in genes like *KRAS* and *TP53* are undruggable.

But there is some hope, even in this perceived “treatment desert”. Recent studies have shown that a subgroup of pancreatic cancers harbor mutations in genes implicated in DNA damage response such as genes of the *BRCA* family and microsatellite instability genes. For these genetically defined small subgroups of PDAC comprising approximately 5% of the neoplasms therapy with PARP inhibitors and checkpoint inhibitors have shown to be effective.

Apart from these unfortunately small molecular subgroups another interesting stratifying approach will likely come into play in the clinical management in PDAC in the near future. Although mutational profiles are homogenous, recent data from several groups proposed that PDACs can nevertheless be classified into distinct molecular groups based on their RNA expression profiles. On this basis, initially, Collisson and colleagues proposed three distinct PDAC types – classical, quasi-mesenchymal and exocrine, which were defined by microarray-based transcriptomic analysis from microdissected tumor cells [2]. Subsequent works by Moffitt et al. [3], and Bailey et al. [4] have both supported and challenged this view and proposed related but yet different classifications. In the Moffitt work, tumors were separated into basal-like and classical neoplasms. Baily proposed four groups, including an immunogenic, a squamous (from the morphology viewpoint a somewhat problematic term since these tumors not necessarily show a squamous differentiation), a progenitor and an ADEX like subtype. Although this sounds confusing at first glance, TCGA has recently put some efforts into investigating the overlap between these classification algorithms [1]. They were able to show that all three approaches quite homogenously delineate a tumor group with QM/basal/squamous molecular features, while all other subgroups were not yet stably reproduced and thus the remaining cases are currently subsummized under the label of “classical” PDAC.

The question of how many of these subgroups will finally be deemed stable seems important, but the clinically paramount question in this context will clearly be, if and how these subgroups impact on disease course and response to therapies, and ultimately, whether the respective algorithms could be used to stratify patients in a clinically useful way. What we do already know is that patients with PDAC belonging to the QM/basal/squamous subtype have an even worse prognosis than the already grim prognosis of the overall PDAC population [2-6]. In addition, there are first hints that in stage IV patients this subtype benefits less from FOLFIRINOX treatment [6,7]. On the other hand, preclinical evidence suggests that certain drugs/drug combinations might work better in specific subgroups including QM/basel/squamous tumors [5]. Along this line, Andricovich and colleagues recently showed that loss of KDM6A might be a key inducer of the squamous like differentiation in this subtype and, even more important, that this can be reverted by BET inhibition, thus defining a potential novel treatment strategy for this deleterious PDAC group [8].

Although the novel subtyping approaches until now have only resulted in our ability to separate a group of patients with an even worse than average prognosis in PDAC for which clearly new treatment strategies must be developed, we believe that assessing the clinical relevance of molecular PDAC subtypes in different treatment scenarios is paramount to move the field forward. This is, however, hampered by technical issues, since RNAseq approaches in the clinical setting are generally complicated to implement. Worse so, in PDAC microdissection (or at least virtual microdissection [3]) would be necessary since stromal RNAseq signatures overcloud tumoral expression profiles making a reliable stratification on the basis of unprocessed bulk RNAseq data highly challenging. Thus, novel bioinformatic strategies (e.g. by developing robust algorithms for virtual microdissection of bulk RNAseq data) or surrogate markers for certain subtypes are clearly necessary to move this concept into clinical application. Taking the latter approach, we have recently suggested a dual combination of Keratin 81 and HNF1α immunohistochemistry to identify PDAC subtypes in an easy, reliable and cost-effective way [6]. Along the same line, data from the COMPASS trial group recently suggested in situ hybridization for GATA6 RNA expression as another potential alternative surrogate for the QM/basal/squamous subtype [7], however all these markers await firm validation and further refinement. And in general, until now, PDAC stratification recapitulating RNA-subtypes, regardless of the methodology used must be viewed rather as biological but not clinical subtyping.

In conclusion, we believe that although pancreatic cancer marches to a different – slower – beat than other large tumor entities when it comes to the personalization of cancer treatment, initial breakthrough discoveries have now been made, which should allow us to move this concept at least a small step forward on the long way to an effective individualized patient treatment even for this devastating disease.
